# Deficient Spindle Assembly Checkpoint in Multiple Myeloma

**DOI:** 10.1371/journal.pone.0027583

**Published:** 2011-11-23

**Authors:** Elena Díaz-Rodríguez, Stela Álvarez-Fernández, Xi Chen, Bruno Paiva, Ricardo López-Pérez, Juan Luis García-Hernández, Jesús F. San Miguel, Atanasio Pandiella

**Affiliations:** 1 Instituto de Biología Molecular y Celular del Cáncer, CSIC-Universidad de Salamanca, Salamanca, Spain; 2 Servicio de Hematología, Hospital Universitario de Salamanca, Salamanca, Spain; Baylor College of Medicine, United States of America

## Abstract

Multiple myeloma (MM) is a hematological disease characterized by an abnormal accumulation of plasma cells in the bone marrow. These cells have frequent cytogenetic abnormalities including translocations of the immunoglobulin heavy chain gene and chromosomal gains and losses. In fact, a singular characteristic differentiating MM from other hematological malignancies is the presence of a high degree of aneuploidies. As chromosomal abnormalities can be generated by alterations in the spindle assembly checkpoint (SAC), the functionality of such checkpoint was tested in MM. When SAC components were analyzed in MM cell lines, the RNA levels of most of them were conserved. Nevertheless, the protein content of some key constituents was very low in several cell lines, as was the case of MAD2 or CDC20 in RPMI-8226 or RPMI-LR5 cells. The recovery of their cellular content did not substantially affect cell growth, but improved their ability to segregate chromosomes. Finally, SAC functionality was tested by challenging cells with agents disrupting microtubule dynamics. Most of the cell lines analyzed exhibited functional defects in this checkpoint. Based on the data obtained, alterations both in SAC components and their functionality have been detected in MM, pointing to this pathway as a potential target in MM treatment.

## Introduction

Multiple myeloma (MM) is the second most frequent hematological disease affecting mainly elderly individuals. It represents 1% of all the neoplasias and 13% of the hematological malignancies [1], [2]. Recently and thanks to new therapies, an increase of survival above 50% has been achieved. Nevertheless it is still a non-curable disease since sooner or later relapses will occur [3]. For that reason, important efforts are being made to identify new therapeutic targets that can be used to treat myeloma patients.

At the cellular level, MM is a B cell neoplasia that affects the last stages of lymphoid differentiation. Three key features of this disease, and critical for its diagnosis, are the accumulation of plasma cells (PCs) in the bone marrow, the production and secretion of immunoglobulins and cytokines, and the activation of osteoclasts that induce bone destruction [4], [5]. In addition, another important characteristic of MM is its highly unstable genome, in which, not only translocations, but also whole chromosome gains and looses have been described. Thus, chromosomal gains have been described in 30% of MM, affecting mainly odd chromosomes and being associated to the hyperdiploid phenotype, in which primary translocations of the immunoglobulins are infrequent [6], [7]. Besides, chromosome 13, in which the human Retinoblastoma gene is located, is frequently lost [8]. Moreover, the presence of these chromosome abnormalities correlates with disease outcome [9], [10], [11].

Chromosomal instability is an important characteristic not only of MM, but also of solid tumors [12], [13]. If aneuploidy is cause or consequence of the tumoral process has long been discussed. Nevertheless, recent reports have demonstrated that aneuploidy generation by the manipulation of proteins involved in the mitotic regulation, such as the components of the spindle assembly checkpoint (SAC), is enough to induce tumor formation in animal models [14], [15], [16], [17]. The SAC is a highly conserved signal transduction pathway that during mitosis controls the adequate distribution of the genomic complement between the two daughter cells. Thus, at the beginning of mitosis, a number of proteins will complex together and localize to the kinetochores blocking cell cycle progression until all the chromosomes are bipolarly attached to the spindle microtubules [18]. Alterations in those proteins will produce abnormal distribution of the chromosomes to the two daughter cells and aneuploidy generation that will eventually lead to tumor formation, as we have previously demonstrated for the SAC proteins MAD2 and HEC1 [14], [16].

From the therapeutic point of view, in the last few years several drugs interfering with SAC function have been investigated and some of them are already being tested in clinical trials (reviewed in [19]). That is the case of inhibitors of Aurora kinases, Polo-like kinases (PLK) or CENP-E. If SAC is altered in MM, it appears reasonable to test the value of these inhibitors in the myeloma clinic. In fact, several recent reports indicate that inhibitors of the mitotic Aurora kinases induce apoptosis in MM cells and could be useful in MM treatment [20], [21], [22], [23], [24]. Also PLK inhibitors could have potential anti-tumor activity in MM [25].

Given the highly unstable karyotype found in MM cells and the lack of knowledge of the status of SAC components in this disease, we wanted to investigate the amount and status of SAC components in MM in order to determine if such checkpoint could have a role in the generation of the aneuploidy observed in this disease.

## Materials and Methods

### Reagents and immunochemicals

Cell culture media, sera, G418 and CellTracker™ red CMPTX were purchased from Invitrogen, Immobilon P membranes from Millipore Corp, and nocodazole from Sigma Chemical Co. Other generic chemicals were purchased from Sigma Chemical Co., Roche Biochemicals or Merck.

The anti-MAD2 and anti-BUBR1 antibodies were from BD-biosciences, the anti-CDC20, anti-BUB3 and anti-KNTC1 from Santa Cruz Biotechnology, the anti-αtubulin from Oncogene Research Products and the anti-PTTG was a generous gift from Dr. Pintor Toro (Andalusian Center for Molecular Biology and Regenerative Medicine, Seville, Spain). The horseradish peroxidase (HRP)-conjugated secondary antibodies were from Bio-Rad and the fluorochrome conjugated ones from Thermo Scientific (Pierce: Dylight).

### Cell lines, cell culture and Western Blot

Multiple myeloma cell lines have previously been described: MM1S, MM1R, MM144, RPMI-8226 U266, OPM2 and NCI-H929 [26]; RPMI-LR5, U266-DOX4 and U266-LR7 [27]. MGG and SJR were derived in the laboratory from patient samples. The use of those clinical samples for investigation was approved by the Ethical Committee of the University Hospital of Salamanca and patients gave their written consent for that use. All those cell lines were grown in RPMI 1640 medium supplemented with 10% fetal bovine serum (FBS) and antibiotics. Previously described 293T [26], [28] and HeLa [29], [30], [31] cells were grown in DMEM supplemented with 5% FBS. All the cell lines were cultured at 37°C in a humidified atmosphere in the presence of 5% CO_2_-95% air.

To prepare cells for protein analyses, they were collected and centrifuged at 10,000 g for 2 minutes, washed in PBS and lysed in ice-cold lysis buffer as described elsewhere [32]. Protein amount was quantified by the Bradford assay and proteins detected by Western blot following standard procedures. For quantitative Western blot, fluorochrome-labeled secondary antibodies were used and the membranes were scanned and analyzed in the Odyssey Infrared Imaging System (Ly-cor Biosciences) using the Odyssey 3.0.16 software.

### Cell cycle synchronization and DNA analysis by flow cytometry

In order to synchronize cells at the G2/M transition, nocodazole treatment was performed. Cells were treated with 200 ng/ml nocodazole for 20 hours and harvested by centrifugation. For DNA analysis by flow cytometry, ethanol fixed cells were stained with 5 µg/ml propidium iodide (PI) and 250 µg DNAse-free RNAse. A total of 30,000 cells were acquired in the PI gate by using a FACSCalibur flow cytometer and CELLQUEST software (Becton Dickinson) and analyzed on MODFIT LT™ software (Verity software).

### Transfections, generation of retroviruses and infection

Transfections and retroviral generation were done as previously described [26], using a modified protocol. Thus 293T cells were transfected using the JetPEI™ reagent (Polyplus-transfection Inc, Illkirch, France) following the manufacturers' instructions. The transfection mix contained DNA of the different plasmids (2.5 µg pMDG-VSV, 5 µg pNGUL-MLV-gag-pol and, 3 µg of the retroviral vector (empty vector or MSCV-IG-hMAD2)) and added to the cells for 8–10 hours. 24 hours after the transfection, medium was replaced with complete virus-collecting medium and 8 hours later, target cells were resuspended in the virus-containing medium including 6 µg/ml polybrene at a concentration of 500,000 cells/ml. Cells were infected three times with 10–12 hours interval between infections.

### Cell proliferation assays

The analysis of MM cell proliferation was performed using a modified methylthiotetrazole (MTT) colorimetric assay that has been previously described [32]. Briefly, cells were plated in triplicates and on the day of the experiment, MTT was added to the wells at 0.5 mg/ml and incubated at 37°C for 4 hours. The MTT formazan crystals were then dissolved in isopropanol-HCl and the absorbance of the samples was recorded at 570 nm using a Tecan fluorimeter with the X-Fluor 4 software. At least three wells were analyzed for each condition, and the results are presented as the mean +/− SD of a representative experiment repeated at least twice.

### Chromosome preparations, cytogenetics and CGH arrays analysis

To count anaphases and visualize their chromosomes, cells were plated on poly-L-lysine-coated glass coverslips. 24–48 hours after plating, cells were fixed in 2% *p-*formaldehyde for 30 minutes, washed in PBS and stained with DAPI. Once mounted, anaphases were counted and analyzed in a Zeiss Axioplan2 fluorescence microscope and representative images acquired by confocal immunofluorescence microscopy using an SP5 Leica apparatus with the LAS-AF software.

For the cytogenetic analysis, MM cells were harvested after mitotic arrest using colcemid (Invitrogen) and metaphase slides were prepared according to standard protocols. Twenty to fifty metaphases were analyzed for each cell line, and the karyotypes were described according to the International Conventional System for Human Cytogenetic Nomenclature (ISCN).

For the CGH arrays, all cell lines were analyzed using a 12×135 K NimbleGen Human CGH Whole Genome v2.0 array (Roche Diagnostics, Mannheim, Germany). This microarray platform provides measurements from 135,000 unique genomic loci. The hybridization, image analysis, extraction of fluorescence intensities and their log_2_ ratios, together with their subsequent normalization, were carried out following the manufacturer's recommendations. Gender-matched human DNA was used as reference. Each of the two reference samples, one female and one male, consisted of a pool of DNA from normal lymphocytes from several healthy people (G147A-G152A; Promega, Madison, WI). Briefly, 500 ng of tumor and reference DNA were labeled with Cy3 and Cy5, respectively. They were combined (4 µg each) and dried by vacuum centrifugation. The DNA was resuspended in 3.3 µL of Sample Tracking Control and vortexed; 8.7 µL of NimbleGen hybridization solution was added to the tube, mixed well, and heated at 95°C for 5 min in the dark. Samples were hybridized for 16–20 h at 42°C, then washed with NimbleGen wash buffers and scanned at 10-µm resolution using the GenePix 4000B dual scanner (Axon Instruments, Burlingame, CA, USA).

Data were extracted from scanned images using NimbleScan 2.5 extraction software (Roche NimbleGen, Madison, WI), which enables automated grid alignment, extraction, and generation of data files. Cy3 and Cy5 images were scanned independently through two separate channels and the quantified data were analyzed using the Cy5/Cy3 intensity ratio converted into log_2_. All the common analysis, visualization and integration steps of array-CGH (aCGH) computational analysis were using waviCGH software [33].

### RNA purification and quantitative RT-PCR

RNA was isolated from the different cell lines as described elsewhere, 1 µg primed with poly-T and cDNA synthesized with M-MLV reverse transcriptase following the manufacturers' instructions (Promega). The relative levels of gene expression of the SAC proteins were determined by real-time quantitative polymerase chain reaction (qRT-PCR) using the oligonucleotides shown in table 1. The reactions were performed using iQTM SYBR Green Supermix (Bio-Rad Laboratories, Madrid) and recorded with the Bio-Rad iQ5 software. The relative quantitation of the gene expression was performed through the cycle threshold (CT) increment method [34]. The results were normalized using GAPDH as a reference gene, and are shown in a heat map. For it, the gplot package for R environment was used with the “Euclidean” method for distance calculation and the complete linkage method for hierarchical clustering.

**Table 1:** Proteins of the SAC tested in MM cell lines.

GENE	OLIGO SENSE	OLIGO ANTISENSE
Aurora K A	AACCTCTGCTTCCTGGGTTT	ACGTTTTGGACCTCCAACTG
Aurora K B	GGGAGAGCTGAAGATTGCTG	GCACCACAGATCCACCTTCT
Aurora K C	ATTTCATTGTGGCCCTGAAG	ATACAGGCGCAGGATATTGG
Borealin	CCTGACACCCAGGTTTGACT	CACTGGCACAGTGAGGAAGA
BUB1	CCTTTGGAGAACGCTCTGTC	TGTGAAGTCTCCTGGGCTCT
BUB3	GGTGGTTCTGATGGCTTTGT	GCAAGCGTAGTCCCATCATT
BUBR1	AGCCAGAACAGAGGACTCCA	ATCCATGGCTGGACTGTAGG
CDC20	CTACAGCCAAAAGGCCACTC	CCAATCCACAAGGTTCAGGT
CENP-E	GTTGATCTTGCAGGCAGTGA	TGAAACCACCAACTTGTCCA
HEC1-NDC80	AGGCAAAGAAGCGATTGAAA	ACCAGCCTCGGGATTAAACT
KNTC1	ATTTCTCCTCCCGTGGATCT	GTTCTGTGCTGTGCCAAAGA
MAD1	CAAGGAGGTTTTCCAGACCA	GCCTTGAAGATGAGGCAGTC
MAD2	GATGACAGTGCACCCAGAGA	TTCCAACAGTGGCAGAAATG
MPS1-TTK1	CAGCAGCAACAGCATCAAAT	TGCTTGAACCTCCACTTCCT
PLK1	GACAAGTACGGCCTTGGGTA	GTGCCGTCACGCTCTATGTA
PLK2	AAAGTTGGGGACTTCGGTCT	CACAGCCATGTCCTTGTTTG
PLK3	CACTTTGAGGACGCTGACAA	GATCTGCCGCAGGTAGTAGC
PLK4	CCACAGACAACAATGCCAAC	GGTCTGCAAATGGAAAAGGA
PTTG-Securin	AAAGCTCTGTTCCTGCCTCA	GAGAGGCACTCCACTCAAGG
Separase	GCCCAGTTACACCTGGAAGA	ACACCCTTGGTCACCTTCTG
Survivin	TGGCAGCTGTACCTCAAGAA	AATCAGGCTCGTTCTCGGTA
ZW10	CGCACAAAGAGGAGAAGACC	CTTCAGCAGCATCTGACCAA
GAPDH	TGCACCACCAACTGCTTAGC	GGCATGGACTGTGGTCATGAG

A list of the SAC and kinetochore genes whose expression levels have been determined in MM cell lines is listed. Besides, the sense or antisense oligonucleotides used to assess such expression are shown.

## Results

### Multiple myeloma cell lines are highly aneuploid

**Figure 1:**
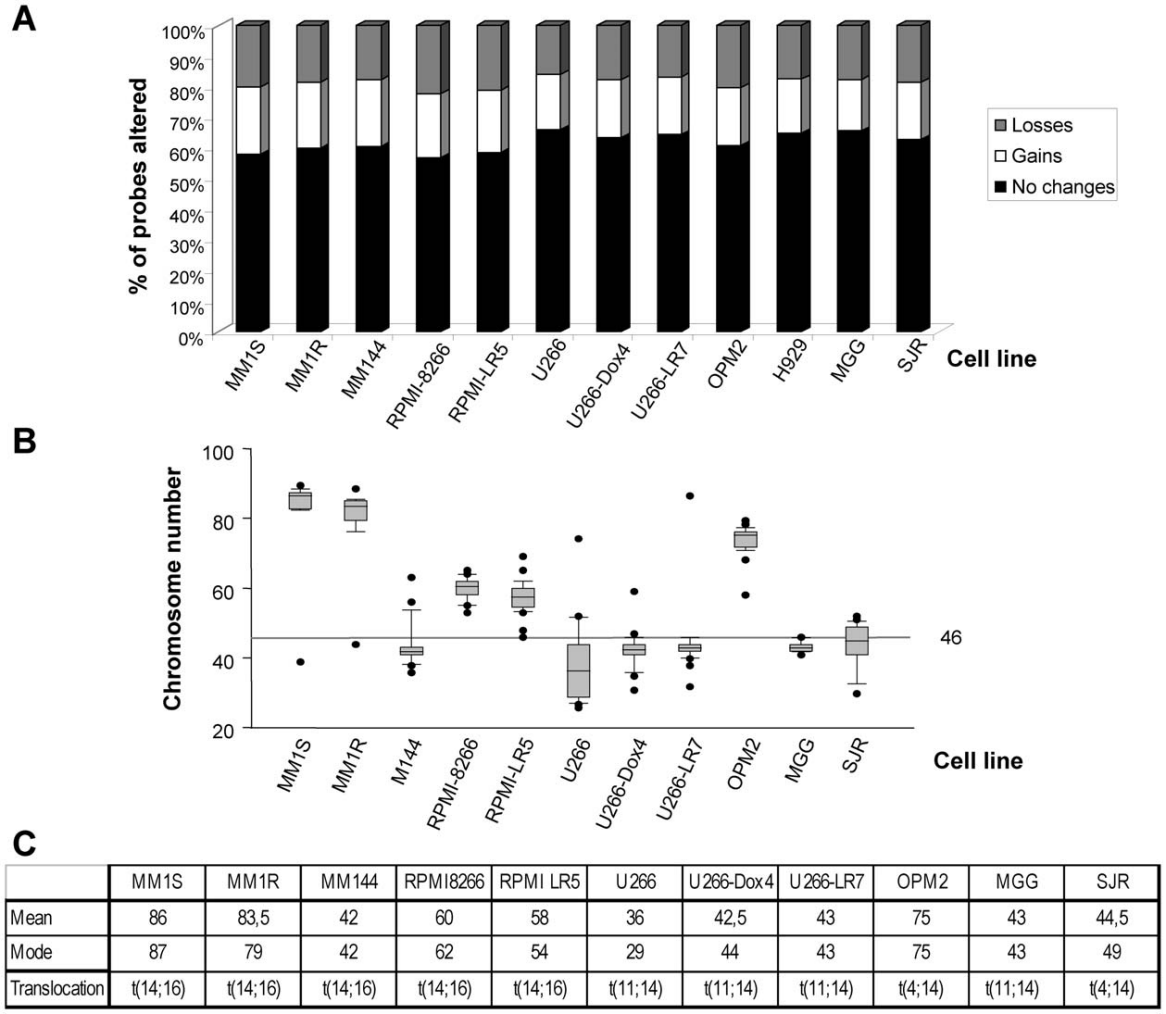
MM cell lines are genetically unstable and typically aneuploid. A. Representation of the karyotype obtained by CGH arrays on genomic DNA of the indicated cell lines. The percentage of the total number of probes that are conserved, gained or lost are indicated in different colors (shown on the right). B. Representation of the karyopype obtained with conventional metaphase chromosome counts. C. Mean chromosome numbers as well as the mode of 50 metaphases counted in the indicated cell lines are shown. Besides, the characteristic translocation found on each cell line is detailed in the bottom row.

One of the characteristics of multiple myeloma (MM) that distinguishes it from other hematological malignancies is the presence of a high number of aneuploidies [5]. In order to analyze the cause of these aneuploidies we first investigated the genetic profile present in different MM cell lines that we had available in our laboratory by using two different and complementary strategies: regular karyotyping and CGH arrays. The analysis of the CGH arrays confirmed that, as expected [35], all the cell lines analyzed were genetically unstable (Figure 1A). Thus, when all the different probes used in this approach were taken into consideration, there was a high number of fragments gained or lost, that was maintained between the cell lines. The percentage of conserved probes varied between 56–65% of the total. In that sense, the most unstable cell lines were RPMI-8226 and MM1S, meanwhile the most conserved one was U266 (Figure 1A). Detailed description of the chromosomes and fragments gained and lost in the different cell lines is shown in Figures S1, S2, S3, S4.

The chromosomal counts obtained with the regular karyotyping confirmed these data (Figures 1B and C). They also showed that OPM2, MM1S and MM1R are mainly tetraploid and, overall, all the MM cell lines analyzed are aneuploid with chromosomal numbers far from the 46 normal diploid content.

### Determination of SAC components mRNA levels in MM cell lines

**Figure 2:**
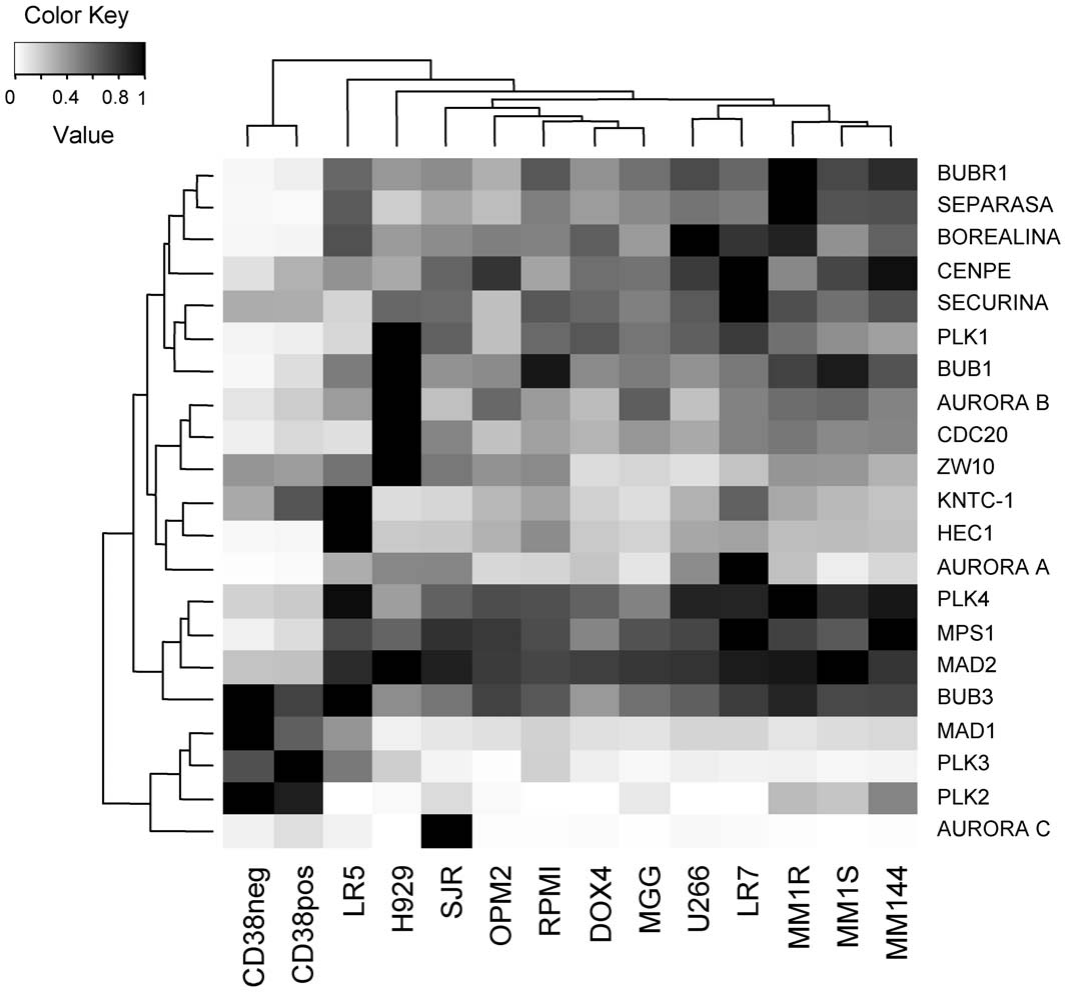
Expression of SAC proteins in MM cell lines. Total RNA purified from the different MM cell lines as well as plasma cells (CD38pos) or other white blood cells (CD38neg) was converted into cDNA by the reverse transcriptase reaction using oligo-dT. Quantitative PCR reaction was performed with oligonucleotide pairs specific for the indicated proteins (indicated on the right) on the different cell lines (shown on the bottom). The relative amount of the different proteins were normalized with the housekeeping gene GAPDH and represented in a heat map.

Several potential causes have been postulated in aneuploidy generation, being one of them the alteration of the SAC. To analyze the potential role of SAC missregulation in the generation of the aneuploidies typical of MM, we determined the levels of most of the proteins that participate in SAC regulation in myeloma cell lines, as well as some other proteins constituent of the kinetochore, and also linked to that checkpoint. For these analyses, RNA from the different exponentially growing cell lines was purified, and quantitative RT-PCR (qRT-PCR) performed. The proteins whose expression has been determined as well as the primers used in this approach are shown in table 1. A total of 23 SAC proteins were analyzed at the RNA level in twelve MM cell lines. As control, the levels of the different proteins were analyzed in plasma cells (CD38+) or other white blood cells (CD38−) obtained and purified from spleen. To normalize the RNA levels, the housekeeping gene GAPDH was used (Figure 2). Overall the levels differed from the two normal cell populations (CD38^+^ and CD38^−^) used as controls, which clustered together (Figure 2). mRNA expression was quite uniform in MM cell lines with only some exceptions. Thus, Aurora kinase C levels were uniformly low in most cell lines, with the exception of the SJR cell line that showed very high levels. Similarly PLK2 or PLK3 levels were low except in MM1S, MM1R and MM144 cells (PLK2) or RPMI-LR5 (PLK3). Interestingly, all these proteins exhibited high levels in the two normal populations. By contrast, the remaining SAC proteins were highly expressed in MM cell lines, with the exception of CDC20, PLK1 and PTTG/securin which displayed low levels in the RPMI-LR5 cell line. Together, these results indicate that MM cell lines expressed levels of SAC components different to normal PCs.

### Deficiency of some key SAC proteins in MM cell lines

**Figure 3:**
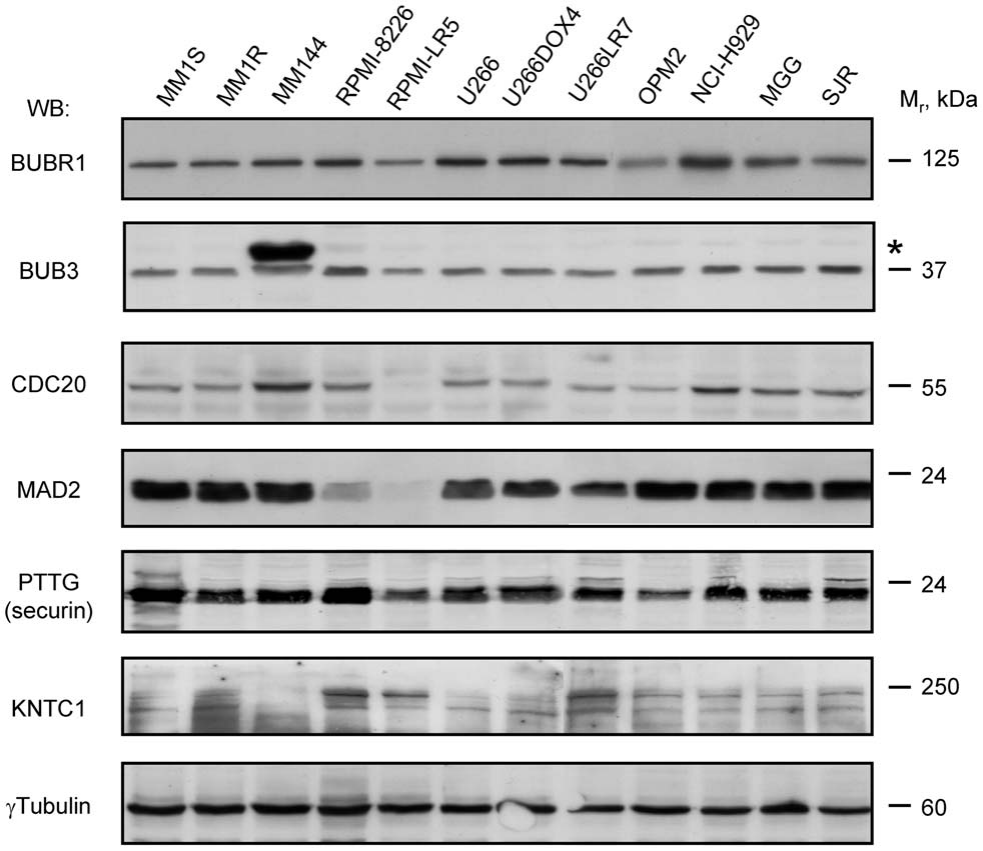
Protein expression of different SAC components in MM. Protein extracts from the MM cell lines indicated at the top of the figure were prepared, quantified, and 50 µg separated by SDS-PAGE. Once transferred, membranes were proved with antibodies specifically detecting the proteins shown on the left.

The protein levels of some key components of the SAC were validated by Western blot (WB) assays. Most of the SAC proteins analyzed were expressed in the different cell lines examined (Figure 3). Besides, as above reported for RNA levels, the protein levels only slightly differed among the cell lines, demonstrating that these levels are tightly controlled. We have also confirmed that some proteins are downregulated, particularly MAD2 in RPMI-8226 and RPMI-LR5 cells or CDC20 in the latter cell line. These cell lines had very low levels of MAD2, but still enough to sustain cellular proliferation. Interestingly, the absolute lack of MAD2 has been reported to be lethal both *in vivo* [36] and *in vitro* [37].

### Reconstitution of MAD2 protein in RPMI cells improves their mitosis

**Figure 4:**
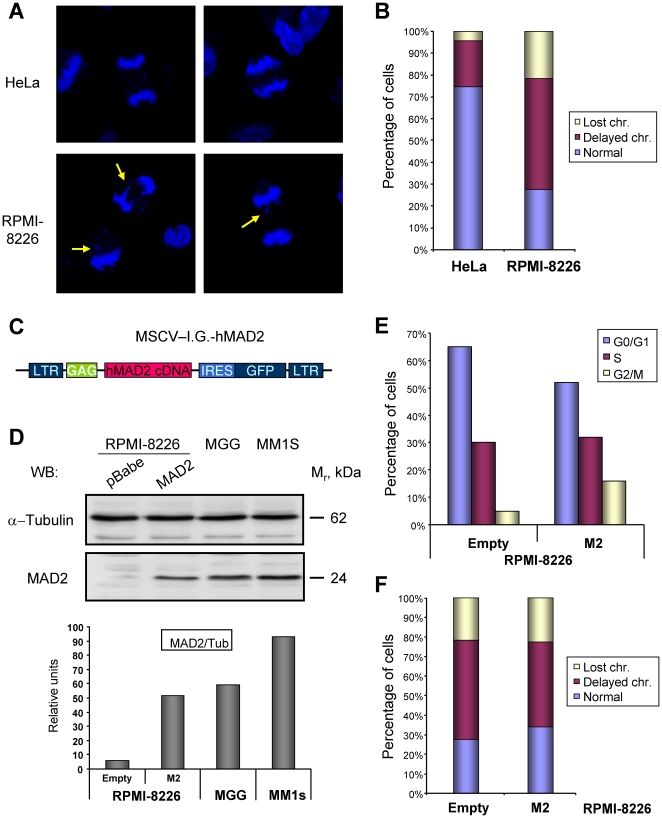
Characterization of the recovery of MAD2 levels on RPMI-8226 cells by retroviral transduction. A. RPMI-8226 cells exhibit chromosome segregation defects. Hela or RPMI-8226 cells were plated on glass coverslips and 2 days later fixed, counterstained with DAPI and anaphases counted at a fluorescence microscope. Yellow arrows point to chromosomal defects. B. The presence of chromosomes left behind (delayed chromosomes) or lost between the two daughter cells was measured in at least 50 anaphases in the indicated cell lines and represented as the percentage of cells with the different defect. C. Schematic representation of the plasmid used to transduce MM cells with hMAD2. D-F. Recovery of MAD2 levels in the MM cell line RPMI-8226. RPMI-8226 cells were transduced with the vector shown in panel C or empty vector, and 2 days later plated for the subsequent analysis. D. Recovery of MAD2 protein levels. Protein extracts from the indicated cell lines were prepared, 30 µg separated by SDS-PAGE and MAD2 and α-tubulin amount determined by regular WB as indicated (upper panels). Another set was similarly analyzed, but their levels were quantified using appropriate secondary antibodies and the Odyssey software. The relative ratio of both proteins is shown in the lower graph. E. The recovery of MAD2 levels induces a small change in their cell cycle profile, producing the accumulation of more cells in the G2/M transition. F. Besides, a small improvement in chromosomal dynamics in mitosis is detected after MAD2 recovery, measured as described in B.

Alteration of MAD2 levels induces chromosome missegregation both *in vitro* and *in vivo* [16], [36], [37]. Given the low levels of this protein in RPMI-8226 cells, chromosomes dynamics were studied. Anaphases were counted both in RPMI-8226 and HeLa cells, an extensively used model cell line with an adequate SAC [30], [31]. In this context, the percentage of abnormal anaphases in which chromosomes were left behind or lost between the two daughter cells were quantified. While HeLa anaphases were close to normal and almost 80% of the anaphases were normal, RPMI-8226 were not as efficient segregating their chromosomes (Figures 4A and B). Thus very frequently chromosomes or DNA fragments were left behind at anaphase and telophase, and chromosomal bridges were also detected (yellow arrows, Figure 4A).

We hypothesized that if those defects in chromosome dynamics were at least partially due to the extremely low levels in MAD2 in this cell line, the recovery in those levels should improve mitosis. To verify this fact, the levels of MAD2 were increased in RPMI-8226 cells by retroviral infection using the vector shown in Figure 4C. Once transduced with this vector, MAD2 levels were analyzed by regular as well as quantitative WB four days later (Figure 4D). Under those conditions MAD2 levels in RPMI-8226 cells infected with the retroviral construct were close to those of the MGG or MM1S cell lines. The moderate increase in MAD2 levels in RPMI-8226 cells did not affect their proliferation, as measured by MTT assay (data not shown), although, as expected based in previous reports [16], [38], it induced a small accumulation of cells at the G2/M transition of the cell cycle (Figure 4E), indicating a more active checkpoint.

When chromosomal dynamics were assessed in those RPMI-MAD2 rescued cells, the distribution of chromosomes between the two daughter cells was slightly improved (Figure 4F), and the percentage of cells with normal mitosis increased, indicating that the missregulation of MAD2 levels could partially cause the genetic instability appreciated in this cell line.

### Most of MM cell lines exhibit a defective SAC

The above results indicated that MAD2 could be involved in controlling the SAC in MM. However the relatively low recovery of SAC function upon restoration of MAD2 suggested that other components of the SAC could be involved in controlling this checkpoint in MM. To test this possibility, we evaluated the functionality of the SAC on MM cell lines expressing normal levels of MAD2. The SAC will detect if at the beginning of mitosis sister chromatids are bipolarly attached to the microtubules of the mitotic spindle by their kinetochores. The presence of a single unattached kinetochore will trigger the checkpoint and block the mitotic progression at metaphase. Thus, in the presence of microtubule-interfering agents, if the SAC is normally functional, cells should be arrested at the G2/M transition without progressing further in the cell cycle. On the contrary, if they lack such checkpoint, they will escape the block.

**Figure 5:**
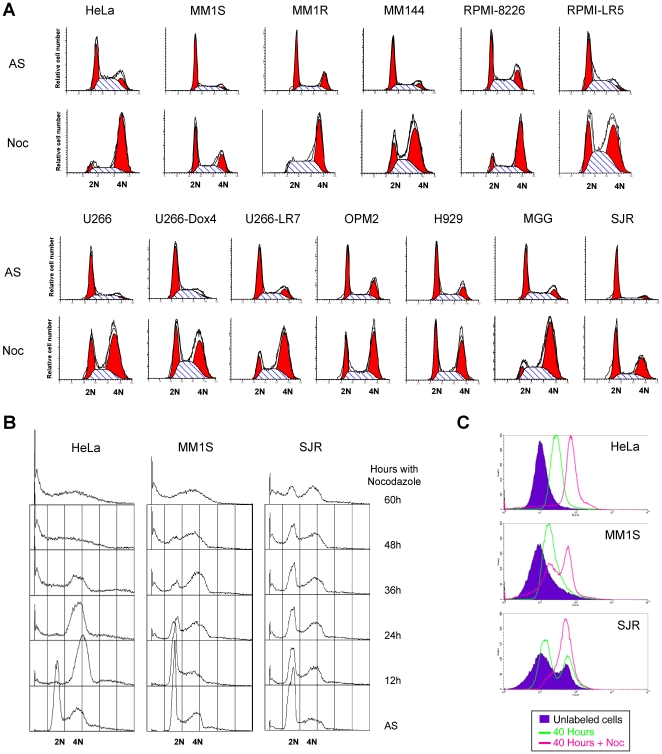
MM cell lines exhibit a deficient SAC when challenged with nocodazole. A. Several MM cell lines were treated with nocodazole for 20 hours, and their cell cycle status after the arrest measured by conventional flow cytometry after DNA staining. Most of the MM cell lines analyzed was not as efficiently arrested in those conditions as HeLa cells. B. The indicated cell lines were arrested for up to 60 hours with nocodazole to determine if the deficient arrest was due to a slower proliferation. The increase in the time of treatment did not effectively synchronize MM1S or SJR cells. C. Cells were labeled for 30 minutes with the CellTracker Red dye, washed out the excess and placed in culture in normal medium (green line), or supplemented with nocodazole (pink line). Cell division in untreated cells made them loose staining, as indicated by the shift in the peak. When the arrest was efficient, the population did not shift (see HeLa cells). Nevertheless, part of the nocodazole treated MM1S population shifted as the control confirming that those cells had, in fact, divided even in the presence of nocodazole. On the other hand the low synchronization detected in SJR was mainly due to the low proliferation of this cell line given that even the normal population had not divided in 40 hours.

To clarify the functionality of the SAC in MM cells, they were challenged with the microtubule-interfering agent nocodazole. For these experiments, and as a well established model of an adequate SAC, we used HeLa cells. In general, normally proliferating MM cell lines exhibited a regular cell cycle profile, even though when compared with HeLa cells, the percentage of cells at the different phases of the cell cycle was shifted towards those in G0/G1 (Figure 5A, upper panels). After nocodazole treatment, the degree of synchronization of MM cell lines was lower than in HeLa cells, even if nocodazole treatment was extended for a longer period (20 hours versus 16 hours for HeLa cells) (Figure 5A). Moreover, some cell lines such as MM1S or SJR did not efficiently arrest under these conditions. This result is important as it indicates that these cell lines escape the SAC control. To verify if such escape was due to a slower proliferation (cells did not reach the checkpoint in 20 hours) or a deficient checkpoint, cells were arrested with nocodazole for longer times and samples were harvested every 12 hours up to 60 hours. Even in those conditions cells did not efficiently arrest (Figure 5B). Instead, after a 36 hour nocodazole treatment they went through cell death, similarly to what happened to HeLa cells.

To confirm that MM cells did escape from the block, we followed another strategy based on the use of fluorescent probes that are retained in living cells. These reagents will be equally distributed into the two daughter cells at cell division, getting each one of them half of the initial markers. MM cells were labeled for 30 minutes with CellTracker Red CMPTX, prior to a 40 hour nocodazole treatment. If cells do escape the arrest a decrease in the staining should be detected. MM1S and SJR together with HeLa (control) cells were investigated. HeLa cells were blocked by nocodazole (Figure 5C, upper panel). By contrast MM1S cells under the same conditions did not efficiently arrest in nocodazole (Figure 5C, 2^nd^ panel). In fact, two subpopulations were found, one that had normally divided and another one that had arrested in nocodazole (Figure 5C). Regarding SJR, it showed a deficient arrest that seem to be due to a low doubling time, as confirmed by the fact that even in the asynchronously growing population part of the cells had not divided after 40 hours (Figure 5C, lower panel).

In conclusion these functional data indicate that MM cell lines have a defective checkpoint when challenged with nocodazole.

## Discussion

One of the characteristics distinguishing MM from other hematological malignances is the presence of a highly abnormal karyotype in which both translocations as well as partial or whole chromosome gains or loses are detected [9], [10]. Looking for the cause of such abnormalities, the spindle checkpoint was analyzed. Using cellular models and RNA expression analyses, the levels of those proteins directly involved in the SAC or the kinetochore dynamics were determined indicating that the core proteins of the SAC or the kinetochore are regularly expressed in MM at the transcription level. Nevertheless, some other proteins further related to the SAC, such as the Auroras (A and C) or PLK2 or 3, show a more variable expression. Thus, for example, the absolute expression level of PLK3 was very low in all the MM cell lines analyzed, except in the line SJR.

Protein levels of most of these genes were also conserved in most of the cells lines analyzed, as demonstrated by Western blotting assays. Nevertheless in the cell lines RPMI-8226 and RPMI-LR5, MAD2 protein was expressed at very low levels. The fact that RNA amount is normal but their protein content is markedly diminished point to either translational defects or decreased stability of this protein in those cell lines. So far, the complete lack of MAD2 protein has been described to be lethal both in cellular [37] and animal models [36]. Surprisingly, RPMI-8226 cells still exhibited a robust SAC function when challenged with nocodazole indicating that they still had a fully active checkpoint independently of MAD2. Nevertheless in this context, the activation of the checkpoint after interfering microtubules dynamics could be due, not only to MAD2, but to another branch of the checkpoint. Thus, it is possible that other known checkpoint protein or unknown MAD2 unrelated protein could replace it in the SAC function in RPMI-8226 cells. Identifying it will help us assign new checkpoint roles in MM. If this is a general mechanism or only applies to MM must also be determined. For example, in the case of BUB1-deficient mouse embryonic fibroblasts, a different response to agents targeting microtubules dynamics has been described [39]: their response to nocodazole is the expected G2/M arrest (causes loss of kinetochore-spindle attachment and tension), but the effect of taxanes is impaired (disrupts tension forces between kinetochores leaving attachment intact). The results that we obtained with nocodazole indicate that MM cells may be resistant to therapies that alter microtubule dynamics, and this is in clear contrast to the efficacy of such therapies in several solid tumors.

Another interesting issue to be investigated is the cause of the defective checkpoint response in MM1S cells. Since all the components of the SAC that we investigated are present, the cellular localization of the whole checkpoint machinery or the presence of mutations in their sequence should be explored. So far, several point mutations in the checkpoint components have been described in solid tumors, some of them affecting the kinases BUB1 and BUBR1. Interestingly, preliminary data from our laboratory (data not shown) demonstrated the presence of several mutations in the kinase BUBR1 in MM1S cells. Some of them, leading to aminoacid change (M40T, Q349R, V618A), have previously been described in solid tumors, such as colorectal cancer, hepatocellular carcinoma or breast cancer, in which they could have a role in impaired checkpoint function [40]. In fact one of them (M40T) affects the KEN domain, a domain responsible for substrate recognition and binding to CDC20 and Blinkin. The potential role of these mutations on BUBR1 functionality as well as the SAC itself must be carefully examined.

MM cells exhibit defects in the SAC either in terms of their components content or its functionality, and therefore it could be used as a target for the development of new therapies for MM. On the other hand, targeted therapies are being developed directed not only to the “core” components but also to other proteins participating in the SAC. Thus, inhibitors of several kinases participating in that checkpoint are under development, and some of them are able to induce apoptosis in MM cells as is the case of Aurora kinase [21], [22], [23], [24], [41] and PLK inhibitors [25]. Moreover, there is a phase II clinical trial recruiting MM patients to be treated with the aurora kinase inhibitor MLN-8237. If the inhibition of other components of the SAC could similarly affect their function, new lines of research could be opened leading to the identification of novel therapeutic targets in the MM field.

## Supporting Information

Figure S1 **Detailed analysis of chromosomes 1–5 by CGH array in the indicated cell lines.** Fragments of the different chromosomes that are gained (right, green) or lost (left, red) are indicated for the different MM cell lines (top of each chromosome). The corresponding chromosome is indicated at the bottom of each panel.
(TIF)

Figure S2 **Detailed analysis of chromosomes 6–11 by CGH array in the indicated cell lines.** Chromosomes and fragments were analyzed and represented as in figure S1.
(TIF)

Figure S3 **Detailed analysis of chromosomes 12–17 by CGH array in the indicated cell line.** Chromosomes and fragments were analyzed and represented as in figure S1.
(TIF)

Figure S4 **Detailed analysis of chromosomes 18–22 by CGH array in the indicated cell lines.** Chromosomes and fragments were analyzed and represented as in figure S1.
(TIF)
