# Supplementary figures and images for: Deficient Spindle Assembly Checkpoint in Multiple Myeloma
Source: PLoS One. 2011 Nov 23;6(11):e27583. doi: 10.1371/journal.pone.0027583 (PMC3223182; doi:10.1371/journal.pone.0027583)

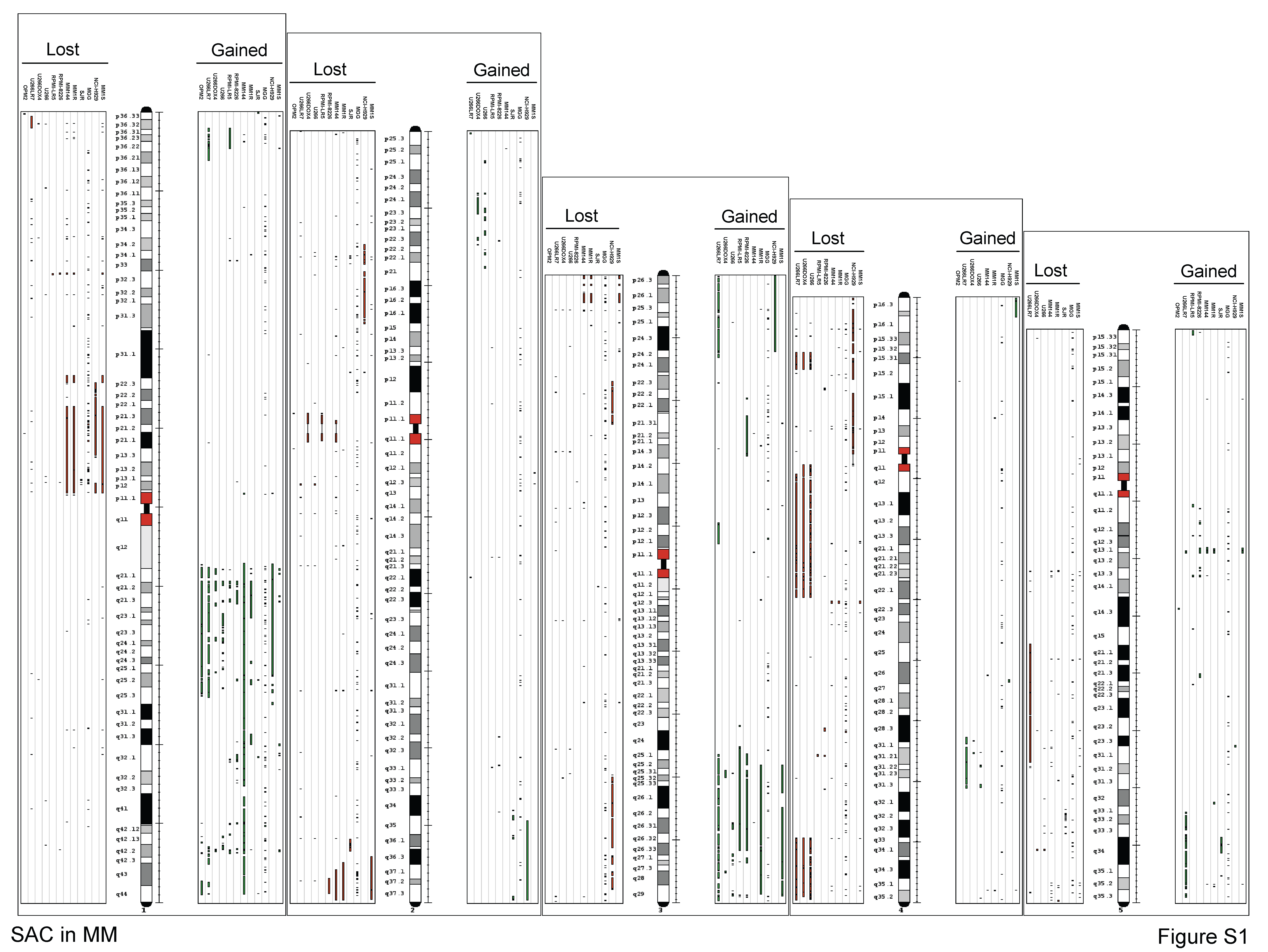

Supplement: Figure S1 — Detailed analysis of chromosomes 1–5 by CGH array in the indicated cell lines. Fragments of the different chromosomes that are gained (right, green) or lost (left, red) are indicated for the different MM cell lines (top of each chromosome). The corresponding chromosome is indicated at the bottom of each panel. (TIF) [file pone.0027583.s001.tif]

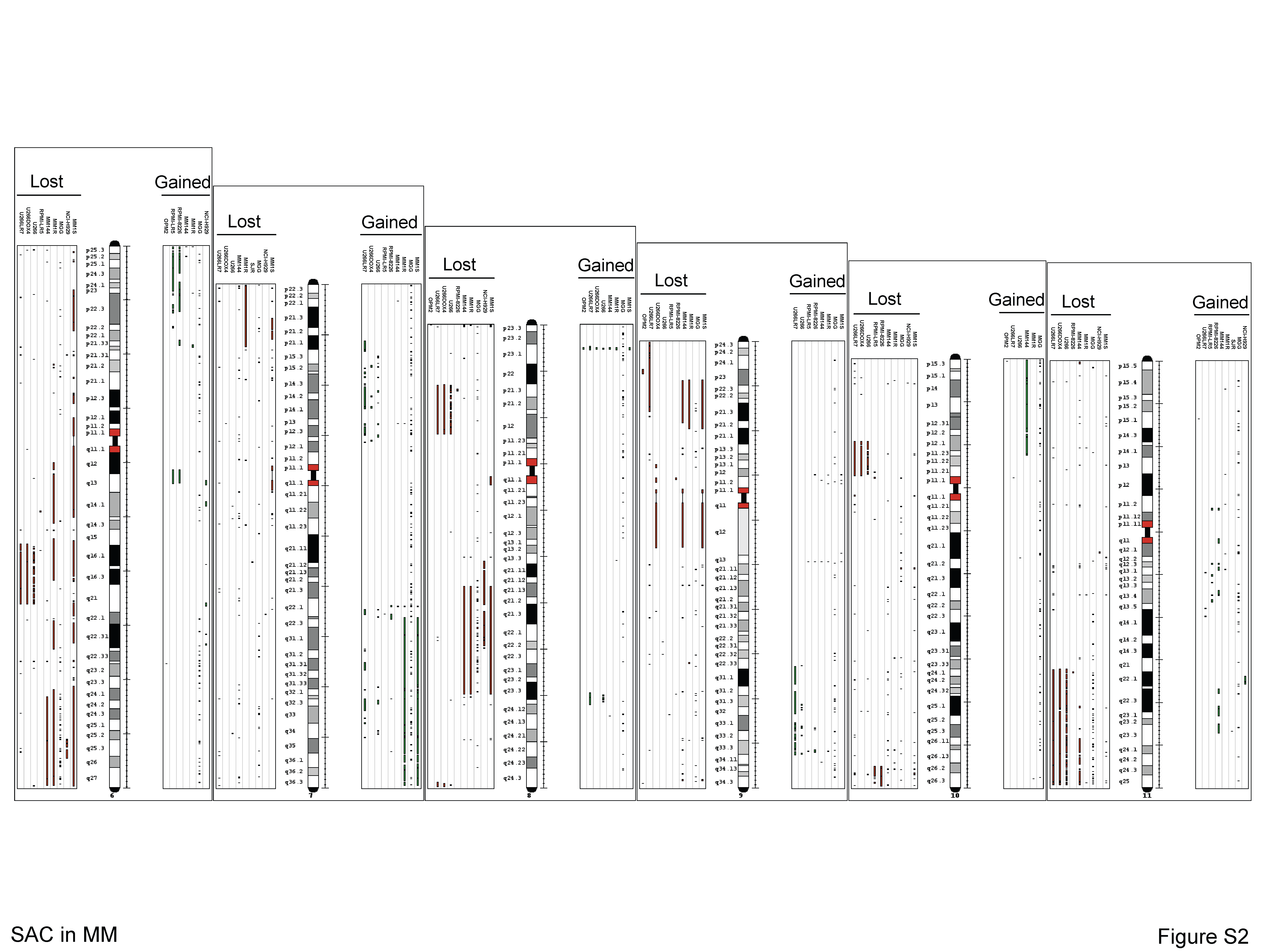

Supplement: Figure S2 — Detailed analysis of chromosomes 6–11 by CGH array in the indicated cell lines. Chromosomes and fragments were analyzed and represented as in figure S1. (TIF) [file pone.0027583.s002.tif]

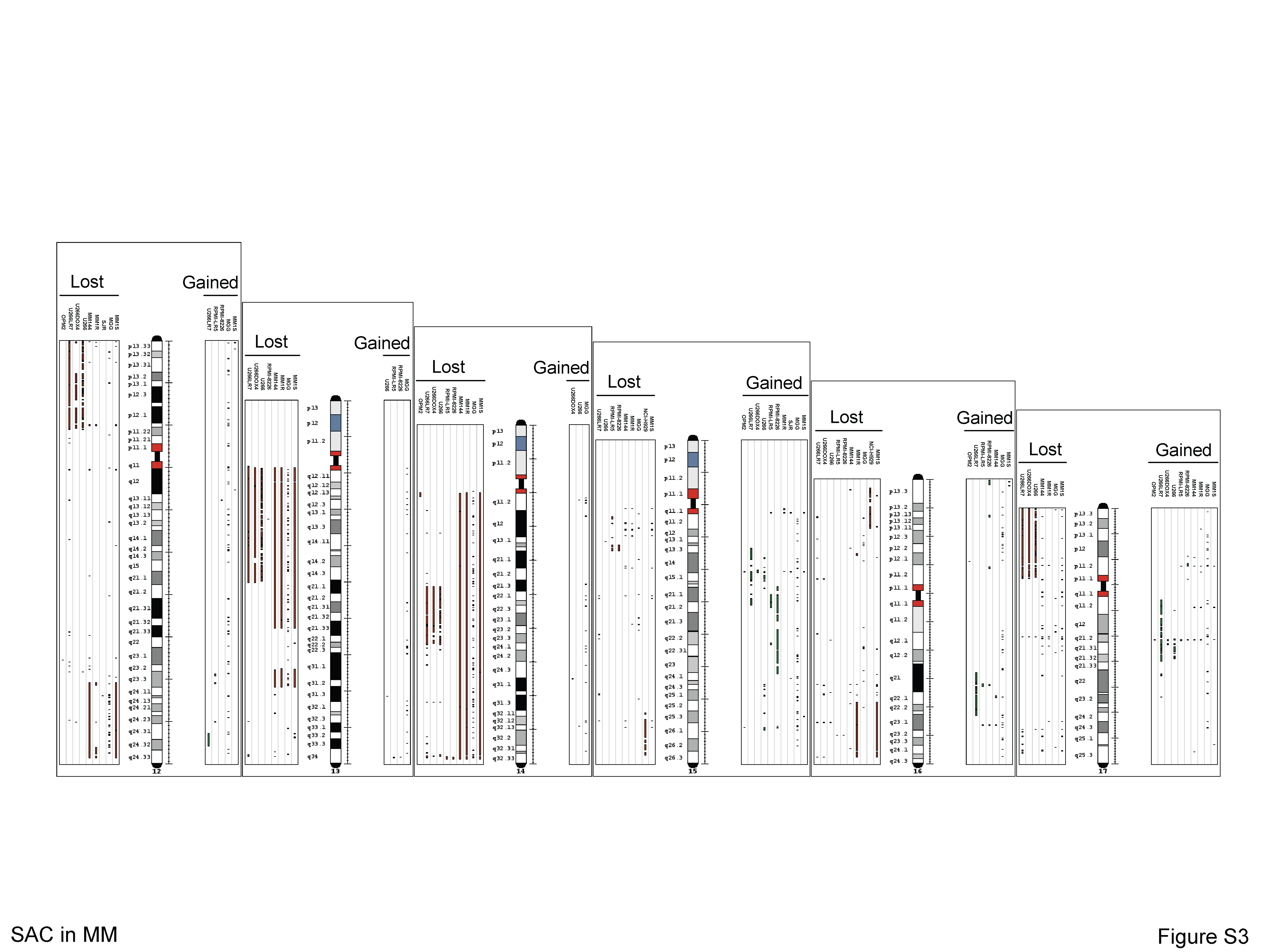

Supplement: Figure S3 — Detailed analysis of chromosomes 12–17 by CGH array in the indicated cell line. Chromosomes and fragments were analyzed and represented as in figure S1. (TIF) [file pone.0027583.s003.tif]

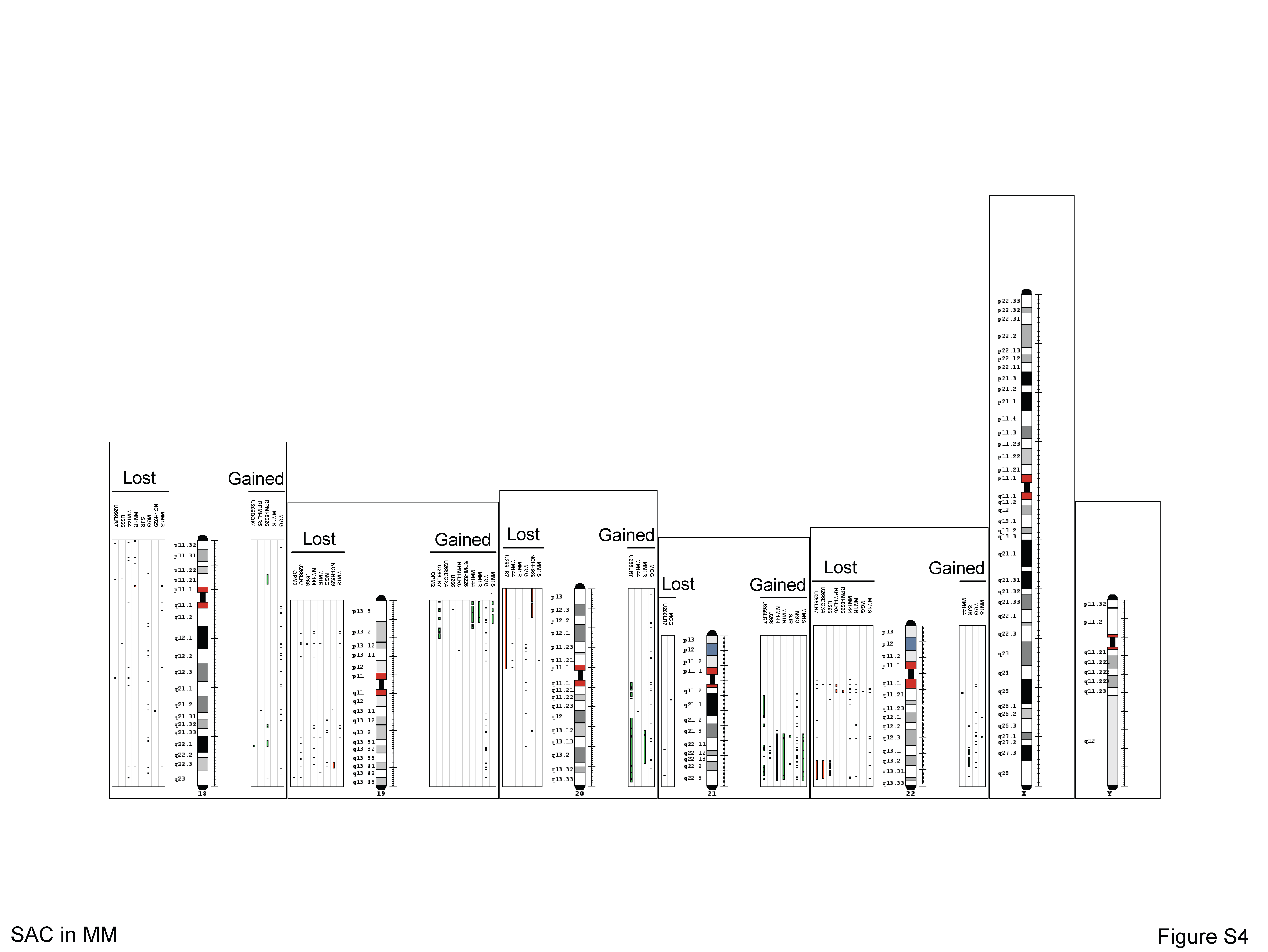

Supplement: Figure S4 — Detailed analysis of chromosomes 18–22 by CGH array in the indicated cell lines. Chromosomes and fragments were analyzed and represented as in figure S1. (TIF) [file pone.0027583.s004.tif]
